# Missed colorectal cancer diagnosis by screening colonoscopy based on the PLCO cancer screening trial

**DOI:** 10.1007/s00384-025-04952-4

**Published:** 2025-10-03

**Authors:** Ying Li, Huan Xiong, Tongzhou Liang, Yuying Liu, Longjun He, Wencheng Tan, Yuhong Wang, Xiaofang Qiu, Bilv Zhong, Chuanbo Xie, Jianjun Li

**Affiliations:** 1 National Cancer Center/National Clinical Research Center for Cancer/Cancer Hospital & Shenzhen Hospital, Chinese Academy of Medical Sciences and Peking Union Medical College, Shenzhen, 518116 China; 2 Department of Endoscopy, State Key Laboratory of Oncology in South China, Collaborative Innovation Center for Cancer Medicine, Sun Yat-Sen University Cancer Center, Guangzhou, 510060 China; 3 Department of Medical Records, The First Affiliated Hospital of Sun Yat-Sen University, Guangzhou, 510060 China; 4 Department of Sports Medicine and Rehabilitation, Peking University Shenzhen Hospital, Shenzhen, Guangdong China; 5 Cancer Prevention Center, State Key Laboratory of Oncology in South China, Collaborative Innovation Center for Cancer Medicine, Sun Yat-Sen University Cancer Center, Guangzhou, 510060 China

**Keywords:** Colorectal cancer, PLCO, Missing rate, Colonoscopy, Screening

## Abstract

**Purpose:**

This study aimed to evaluate the proportion of colorectal cancer (CRC) missed by colonoscopy and the characteristics of the patients with missed diagnosis using data from the prostate, lung, colorectal, and ovarian (PLCO) cancer screening trial, and to analyze and compare patient survival between detection and missed diagnosis groups for an evidence-based basis for improving the effectiveness of colorectal cancer screening.

**Patients and methods:**

Patients with CRC identified by baseline or follow-up colonoscopy and those identified by annual study update questionnaires or National Death Index search, without any positive findings by colonoscopy in the screening arm of the PLCO study, were included in this study. We calculated the rate of missed CRC diagnosis by colonoscopy using the definition of missed cases as the numerator and the sum of patients with CRC as the denominator.

**Results:**

Three hundred sixty patients with CRC were included in the final analysis (detection group, *n* = 298; missed diagnosis group, *n* = 62). The overall rate of missed CRC diagnosis by colonoscopy was 17.22%. Patients with a history of colorectal polyps had a higher rate of missed diagnoses (33.3%). The missed diagnosis rate was higher in patients with proximal CRC (31.3%). CRC occurring in the transverse colon (29.6%), hepatic flexure of the colon (40.0%), ascending colon (27.0%), and cecum (36.6%) were more likely to be missed by colonoscopy. The later the stage, the higher the missed CRC diagnosis rate (10.5, 20.0, 30.8, and 30.8% for stages I–IV, respectively).

**Conclusion:**

Colonoscopy missed a relatively high proportion of CRC, mainly in the proximal colon (especially in the hepatic flexure and cecum of the colon). Recent developments in non-invasive screening technologies, such as stool DNA testing and liquid biopsy, may help address the limitations of colonoscopy. Combining these approaches with traditional endoscopy could enhance overall detection accuracy and reduce the rate of missed colorectal cancer.

## Introduction

Colorectal cancer (CRC) is the first leading cause of cancer death in men and second in women associated with a high number of new cases in 2024 [1]. Early CRC screening strategies have been proven to significantly reduce the morbidity and mortality of this type of cancer [2]. Recent studies have suggested annual fecal immunochemical test and computed tomography colonography as feasible CRC screening strategies [3, 4]. Nonetheless, optic colonoscopy screening remains the gold standard for CRC screening and surveillance [5, 6]. Moreover, early detection and removal of adenomas and precancerous polyps may prevent CRC onset [7]. However, the effectiveness of colonoscopy screening may be impaired because of poor intestinal preparation, limited experience of the operator, different lesion sites, and tumor appearance [8]. A meta-analysis by Zhao et al. reported a miss rate of approximately 26% for adenomas and approximately 27% for serrated polyps [9].

A relatively large proportion of patients with post-colonoscopy colorectal cancer (PCCRC) may be attributed to the missed diagnosis during the previous screening [10, 11]. Previous studies showed that the mortality of PCCRC is higher than that of colonoscopy-detected CRC [10, 11]; thus, reducing the rate of missed CRC diagnosis would substantially improve long-term survival [12]. Hence, identifying the missed diagnosis rate of precancerous lesions and the related risk factors and indicators is crucial.

In this study, we aimed to further explore the proportion of CRC that was missed by colonoscopy and the characteristics of the patients using the prostate, lung, colorectal, and ovarian (PLCO) cancer screening trial database. The distribution of patients with missed diagnosis, CRC characteristics, and survival status was analyzed. This study employed prospective research methods to provide evidence-based results for the improvement in the screening effectiveness of CRC.

## Methods

### Data source

Data were obtained from the National Cancer Institute’s prostate, lung, colorectal, and ovarian (PLCO) cancer screening trial, which is a randomized, controlled trial to determine the effect of certain screening tests on mortality of prostate, lung, colorectal, and ovarian cancer [13]. Briefly, approximately 155,000 participants from 10 PLCO screening centers across the USA between November 1993 and July 2001 were enrolled. At entry, participants were randomly and equally divided into two groups: control and intervention groups. The control group received routine health care from their physicians; the intervention group received screening exams for prostate, lung, colorectal, and ovarian cancers, as outlined in the study protocol. The eligibility criteria for the PLCO cohort were as follows: (1) age between 55 and 74 years; (2) not undergoing cancer treatment other than skin cancer; (3) no known history of prostate, lung, colon, or ovarian cancer; and (4) no colonoscopy, sigmoidoscopy, or barium enema in the past 3 years (applicable to individuals randomized after April 1995).

To obtain research data, we submitted a proposal that includes the proposed research project and the types of data required for the study. After being reviewed and approved by the NCI trial leader and signing the data transmission agreement, the individual can access the necessary data and materials within a limited time (project number: PLCO-459).

### Study population and measures

In the PLCO cancer screening trial, participants in the intervention group were expected to have undergone two colonoscopies: one at baseline (T0) and another at 3 or 5 years thereafter (T3/5). Approximately 107,000 screening colonoscopies (65,000 at baseline and 42,000 at T3 or T5) were conducted among 67,000 participants who had at least one screening colonoscopy, and 39,000 participants underwent colonoscopy both at baseline and during their return visit. The specific time of baseline and follow-up colonoscopy was recorded in the PLCO cancer screening trial database. Participants with abnormalities based on colonoscopy were referred to a general practitioner’s clinic for further colonoscopy and histopathological follow-up. Information was extracted from the endoscopy database, which is part of the PLCO cancer screening trial database and contains records of endoscopy findings, postoperative pathological results, and colorectal cancer diagnoses, coded by designated personnel. In addition to the direct detection of CRC by colonoscopy, the annual CRC incidence was identified by administering the annual study update (ASU) questionnaires to the participants through mail and summarizing the results of the National Death Index (NDI) search. In the PLCO cancer screening trial database, researchers recorded the exact time of CRC diagnosis in patients. Participants with positive colonoscopy findings were referred to their primary care providers for further diagnostic and therapeutic procedures, including endoscopic or surgical treatment as appropriate. However, detailed information on treatment types and clinical outcomes was not available in the PLCO dataset and thus was not analyzed in this study.

To evaluate the incidence of CRC missed by colonoscopy, the subjects in our study were defined as patients with CRC identified by baseline or follow-up colonoscopy and those identified by ASU questionnaires or NDI search without any positive findings by colonoscopy. For this study, missed CRC was operationally defined as: (1) CRC diagnosed within 3 years after a colonoscopy that identified adenoma or advanced adenoma, (2) CRC diagnosed within 7.5 years after identification of hyperplastic or benign polyps, and (3) CRC diagnosed through ASU or NDI in patients with no prior positive colonoscopy findings. We acknowledge that this definition may include some interval cancers that developed de novo rather than being present but undetected at the time of colonoscopy.

**Fig. 1:**
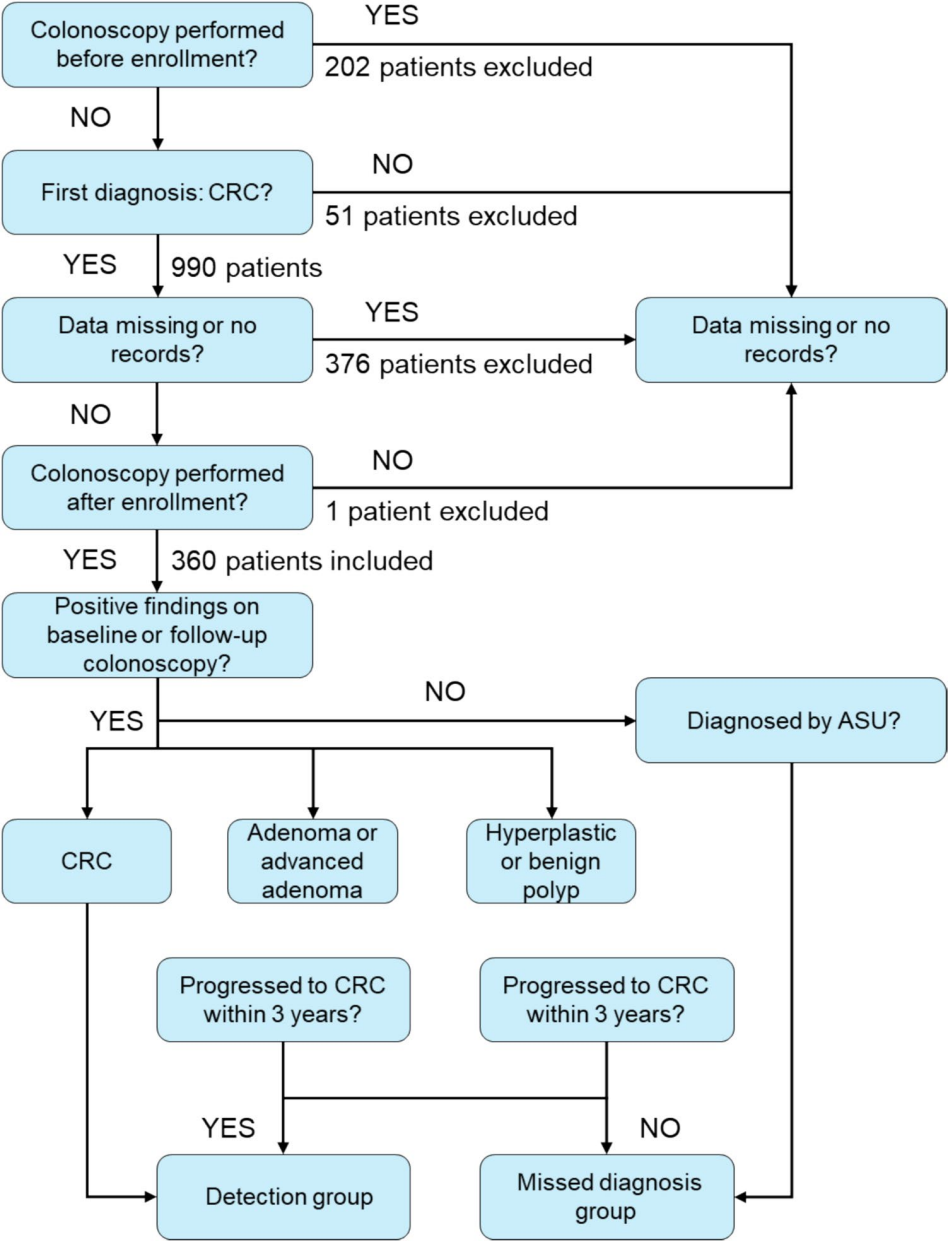
Algorithm used to categorize cancer in the PLCO cancer screening trial. CRC, colorectal cancer; ASU, annual study update

Of the 107,000 participants who underwent screening colonoscopies, 990 were diagnosed with CRC. Of the 990 patients with CRC, 202 patients, who underwent endoscopic examination before enrollment, were excluded. Although this exclusion was not strictly pre-specified in the PLCO study design, it was applied to ensure that only cancers first identified during the screening period were included, avoiding bias from prior diagnostic procedures. Fifty-one patients who were not diagnosed with CRC first (that is, CRC was not the primary malignant tumor but the metastatic tumors of other sites) and 376 patients with CRC with missing data or incomplete records at baseline or follow-up colonoscopy were also excluded. Lastly, one participant, who did not undergo any digestive endoscopic procedure (including colonoscopy, endoscopy, and sigmoidoscopy), was excluded. Thus, a total of 360 CRC patients were included in the final analysis. The process of screening and grouping is illustrated in Fig. 1.

### Statistical analyses

Age was expressed as mean ± standard deviation. The sociodemographic and clinical characteristics of the subjects were described by constituent ratios. We calculated the rate of missed CRC diagnosis by colonoscopy using the aforementioned definition of missed cases as the numerator, while using the sum of patients with CRC identified by baseline or follow-up colonoscopy and by ASU questionnaires or NDI search (without any positive findings by colonoscopy) as the denominator. The chi-square test was used to compare the differences in sociodemographic characteristics, family history, tumor stage, tumor location, and left and right hemicolon cancer among patients with CRC between the missed diagnosis group and the detection group. A logistic regression analysis model was employed to compare the risk of missed CRC diagnosis among subjects with different demographic or clinical characteristics. The log-rank test was used to compare the survival status between patients with CRC detected and those with CRC missed by colonoscopy screening. Inspection level *ɑ* = 0.05. All statistical analyses were performed using SAS 9.3 statistical software (Cary, NC, USA).

## Results

### Comparison of baseline characteristics between the detection and missed diagnosis groups

The baseline characteristics of the patients included in the final analysis were similar between the detection and missed diagnosis groups. No significant intergroup differences in age, sex, race, education, or family history of CRC were found. However, differences in the history of colon polyps, site of cancer (left or right hemicolon), specific cancer location, and cancer stage were noted between the two groups (Table 1).

**Table 1:** Baseline characteristics of patients in the final analysis set

	Detection group Number (%)	Missed diagnosis group Number (%)	Chi-square value	***P*** value
Age at diagnosis, years				
≤ 59	49 (16.4)	13 (21.0)	2.393	0.495
60–64	110 (36.9)	17 (27.4)		
65–69	81 (27.2)	20 (32.3)		
≥ 70	58 (19.5)	12 (19.3)		
Number of subjects (*N*)	298	62		
Sex				
Male	203 (68.1)	38 (61.3)	1.082	0.298
Female	95 (31.9)	24 (38.7)		
Number of subjects (*N*)	298	62		
Race				
White	262 (88.0)	54 (87.1)	0.032	0.857
Nonwhite	36 (12.0)	8 (12.9)		
Number of subjects (*N*)	298	62		
Education				
< High school	33 (11.1)	7 (11.3)	0.111	0.946
High school or equal	112 (37.7)	22 (35.5)		
College or above	152 (51.2)	33 (53.2)		
Number of subjects (*N*)	297 (1 missing)	62		
Family history of CRC				
No	239 (85.1)	50 (87.7)	0.272	0.602
Yes	42 (14.9)	7 (12.3)		
Number of subjects (*N*)	281 (17 missing)	57 (5 missing)		
History of colon polyps				
No	272 (91.9)	50 (80.6)	7.169	0.007
Yes	24 (8.1)	12 (19.4)		
Number of subjects (*N*)	296 (2 missing)	62		
Cancer location/side				
Distal	207 (69.7)	21 (33.9)	28.679	< 0.0001
Proximal	90 (30.3)	41 (66.1)		
Number of subjects (*N*)	297 (1 missing)	62		
Cancer location				
Rectum and anus	83 (27.9)	11 (17.7)	37.858	< 0.0001
Rectosigmoid junction	10 (3.4)	1 (1.6)		
Sigmoid colon	104 (35.0)	5 (8.1)		
Descending colon	10 (3.4)	4 (6.5)		
Splenic flexure of the colon	9 (3.0)	2 (3.2)		
Transverse colon	19 (6.4)	8 (12.9)		
Hepatic flexure of the solon	9 (3.0)	6 (9.7)		
Ascending colon	27 (9.1)	10 (16.1)		
Cecum	26 (8.8)	15 (24.2)		
Number of subjects (*N*)	297 (1 missing)	62		
Cancer stage				
I	153 (55.5)	18 (29.5)	20.218	0.001
II	60 (21.7)	15 (24.6)		
III	45 (16.3)	20 (32.8)		
IV	18 (6.5)	8 (13.1)		
Number of subjects (*N*)	276 (22 missing)	61 (1 missing)		

### Analysis of missed CRC diagnosis by colonoscopy

After screening, 360 patients with CRC were included in the final analysis (detection group, *n* = 298; missed diagnosis group, *n* = 62). Table 2 shows the results of the univariate analysis of the different characteristics of patients with missed diagnosis. The overall rate of missed CRC diagnosis by colonoscopy was 17.22%. Compared with patients without colorectal polyps, those with a history of colorectal polyps had an increased risk of missed diagnosis (odds ratio [OR], 2.72; 95% confidence interval [CI], 1.278–5.791). The risk of missed diagnosis of proximal CRC increased compared with that of distal CRC (OR, 4.49; 95% CI, 2.511–8.03). Compared with CRC in the rectum or anus, CRCs in the transverse colon (OR, 3.177; 95% CI, 1.125–8.972), hepatic flexure of the colon (OR, 5.03; 95% CI, 1.501–16.858), ascending colon (OR, 2.795; 95% CI, 1.07–7.30), and cecum (OR, 4.353; 95% CI, 1.78–10.644) were more likely to be missed by colonoscopy. In addition, the later the CRC stage, the higher the risk of missed diagnosis. Compared with patients with stage I CRC, patients with stages II, III, and IV CRC had a 2.125-fold (95% CI, 1.006–4.487), 3.778-fold (95% CI, 1.842–7.748), and 3.778-fold (95% CI, 1.439–9.92) increased risk of missed diagnosis by colonoscopy, respectively. No significant differences in the risk of missed diagnosis according to age, sex, race, education level, or family history of CRC among first-degree relatives were found.

**Table 2:** Univariate analysis of the different characteristics of patients with missed CRC diagnosis

	Number of subjects (***N***)	Detection group Number (%)	Missed diagnosis group Number (%)	Univariate analysis	
				OR (95% CI)	*P* value
**Age at diagnosis, years**					
** ≤ 59**	62	49 (16.4)	13 (21.0)	1.00	
**60–64**	127	110 (36.9)	17 (27.4)	0.583 (0.263–1.292)	0.184
**65–69**	101	81 (27.2)	20 (32.2)	0.931 (0.425–2.037)	0.857
** ≥ 70**	70	58 (19.5)	12 (19.4)	0.78 (0.326–1.865)	0.576
**Sex**					
**Male**	241	203 (68.1)	38 (61.3)	1.00	
**Female**	119	95 (31.9)	24 (38.7)	1.35 (0.766–2.377)	0.299
**Race**					
**White**	316	262 (87.9)	54 (87.1)	1.00	
**Non-White**	44	36 (12.1)	8 (12.9)	1.078 (0.475–2.448)	0.857
**Education**					
** < High school**	40	33 (11.1)	7 (11.3)	1.00	
**High school or equal**	134	112 (37.7)	22 (35.5)	0.926 (0.364–2.359)	0.872
**College or above**	185	152 (51.2)	33 (53.2)	1.023 (0.417–2.513)	0.960
**Family history of CRC**					
**No**	289	239 (85.1)	50 (87.7)	1.00	
**Yes**	49	42 (14.9)	7 (12.3)	0.797 (0.338–1.876)	0.603
**History of colon polyps**					
**No**	322	272 (91.9)	50 (80.6)	1.00	
**Yes**	36	24 (8.1)	12 (19.4)	2.72 (1.278–5.791)	0.010
**Cancer location/side**					
**Distal**	228	207 (69.7)	21 (33.9)	1.00	
**Proximal**	131	90 (30.3)	41 (66.1)	4.49 (2.511–8.03)	< 0.0001
**Cancer location**					
**Rectum and anus**	94	83 (27.9)	11 (17.7)	1.00	
**Rectosigmoid junction**	11	10 (3.4)	1 (1.6)	0.755 (0.088–6.476)	0.797
**Sigmoid colon**	109	104 (35.0)	5 (8.1)	0.363 (0.121–1.085)	0.070
**Descending colon**	14	10 (3.4)	4 (6.5)	3.018 (0.807–11.288)	0.101
**Splenic flexure of the colon**	11	9 (3.0)	2 (3.2)	1.677 (0.32–8.785)	0.541
**Transverse colon**	27	19 (6.4)	8 (12.9)	3.177 (1.125–8.972)	0.029
**Hepatic flexure of the colon**	15	9 (3.0)	6 (9.7)	5.03 (1.501–16.858)	0.009
**Ascending colon**	37	27 (9.1)	10 (16.1)	2.795 (1.07–7.30)	0.036
**Cecum**	41	26 (8.8)	15 (24.2)	4.353 (1.78–10.644)	0.001
**Cancer stage**					
**I**	171	153 (55.4)	18 (29.5)	1.00	
**II**	75	60 (21.7)	15 (24.6)	2.125 (1.006–4.487)	0.048
**III**	65	45 (16.3)	20 (32.8)	3.778 (1.842–7.748)	0.0003
**IV**	26	18 (6.5)	8 (13.1)	3.778 (1.439–9.92)	0.007

*OR* odds ratio, *CI* confidence interval, *CRC* colorectal cancer.

### Comparison of survival status between the groups

**Fig. 2:**
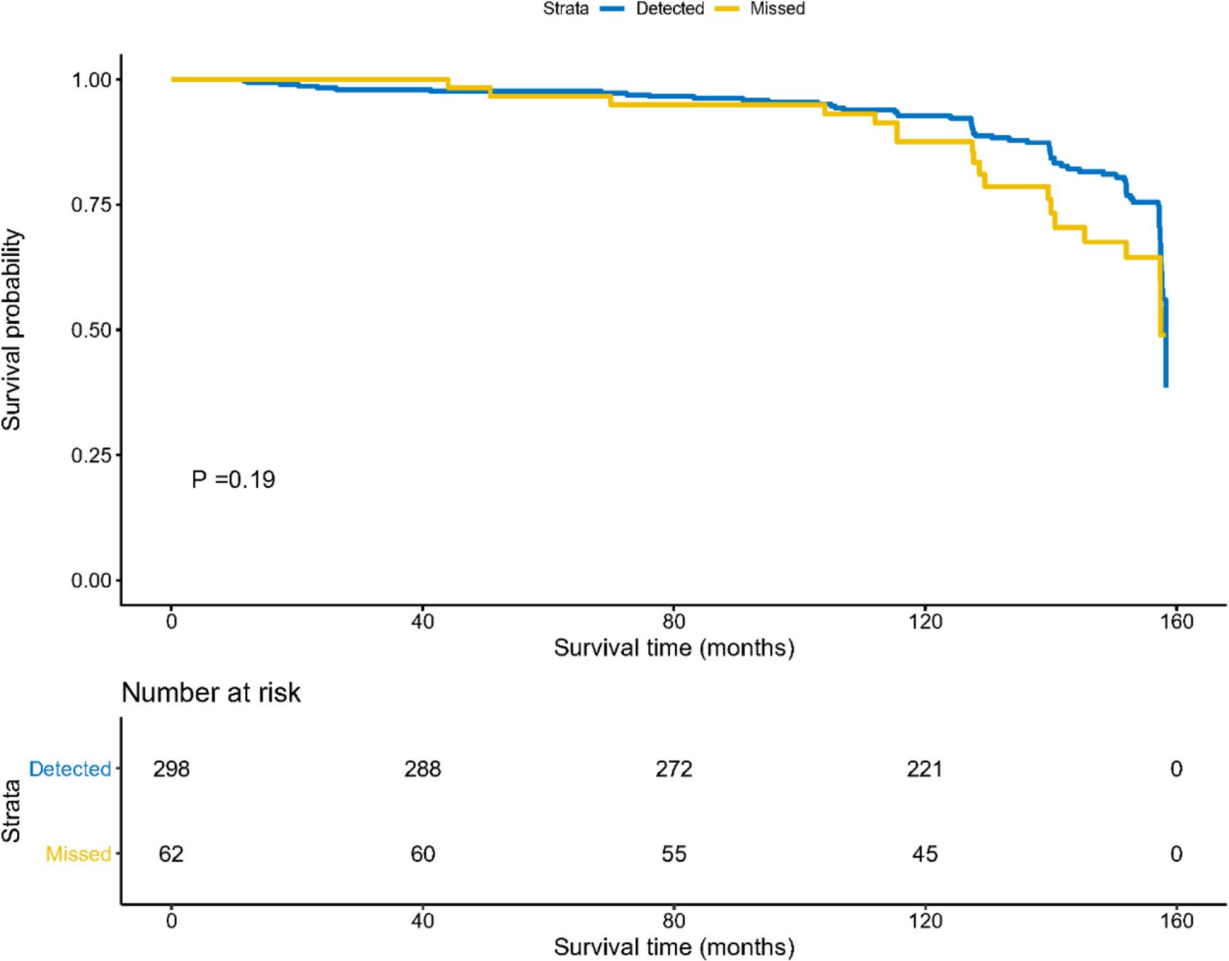
Survival curves of the patients with CRC detected and missed by colonoscopy according to ASU

Figure 2 shows the survival curve of patients with CRC in the detection and missed diagnosis groups. Survival time (in months) was defined as the time from trial entry (randomization) to death or the time from trial entry (randomization) to trial exit. Based on the figure, patient survival was better in the detection group than in the missed diagnosis group; however, the final analysis results indicated no statistically significant difference between the two groups (*P* = 0.19), which could be attributed to the small sample size included in the final analysis.

## Discussion

Using the randomized population-based PLCO cancer screening trial database, we identified that CRC was missed by colonoscopy screening in approximately 20% of the patients, which is significantly higher than the missed diagnosis rate reported by Bressler et al. in 2007 [14], comparable to the adenoma miss rate of colonoscopy reported by Zhao et al. (26%), and close to the rate of missed CRC diagnosis reported by Huang et al. (19.9%) [9]. Although the exact mechanisms underlying missed diagnoses are not fully understood, possible contributing factors include the following:

First, suboptimal bowel preparation can impair mucosal visibility, significantly reducing the ability to detect lesions. Park et al. reported a significantly increased adenoma detection rate in subjects with better intestinal preparation [15]. Second, operator-dependent factors, such as variability in endoscopist expertise and withdrawal technique, may lead to incomplete examination, particularly in hard-to-reach areas. Third, anatomical challenges, such as flat or sessile lesions hidden behind colonic folds, especially in the proximal colon, can result in lesions being overlooked. Finally, the presence of serrated or rapidly progressing lesions with subtle morphological features may contribute to false-negative findings even in technically adequate procedures.

This study found that the rate of missed CRC diagnosis by colonoscopy is higher in patients with a history of colonic polyps. Traditional adenomas have long been recognized as CRC precursors and served as the primary driver of post-polypectomy surveillance guidelines. However, over the past few decades, we have learned that at least 20% of CRCs are caused by serrated polyps [16]. Recently, attention to the risk of metachronous lesions in patients with serrated polyps has been increasing. This suggests that in patients with a history of colonic polyps, we should not only make adequate bowel preparation before colonoscopy but also carefully and meticulously perform the operation to reduce the missed diagnosis rate. In addition, this study found that patients in the missed diagnosis group were more likely to present with advanced-stage CRC at the time of detection, suggesting a possible association between missed diagnosis and delayed disease identification, rather than implying that advanced cancers are inherently more likely to be missed. Compared with early-stage CRC (I/II), advanced stage CRC (III/IV) had a higher degree of malignancy and poorer prognosis. Hence, missed diagnosis of advanced CRC can significantly reduce the overall 5-year survival rate of patients with CRC.

Regarding cancer location, this study found that proximal CRCs are more likely to be missed by colonoscopy than distal CRC. Proximal colorectal cancers were more frequently missed in our study. This may be due to challenges in visualizing lesions in the proximal colon, such as angulated segments and deeper folds, as well as operator fatigue or incomplete intubation. The reasons for the relatively high rate of missed diagnosis of proximal CRC are as follows: First, proximal CRC generally develops at a later age [9]. Previous studies have shown that the rate of missed CRC diagnosis gradually increases with age [17], which could be attributed to the decreasing patient compliance with colonoscopy. Second, with the length of the endoscope and the complexity of endoscopic operation, it is more difficult to reach proximal CRC than distal CRC. Third, given the differences in anatomical structure between proximal and distal CRCs, the clinical stage of patients with proximal CRC is generally relatively late. As previously mentioned, advanced CRC is more likely to be missed. In addition, in proximal CRC, the hepatic flexure of the colon and cecum had the highest risk of missed diagnosis. Moreover, the cecal intubation success rate is a critical quality indicator for complete colonoscopy [18]. Failure to reach the cecum may result in incomplete examination and increase the likelihood of missing proximal lesions, including those in the cecum and ascending colon. Although cecal intubation time was mentioned in previous studies, the PLCO dataset does not provide procedure-level success rate data, limiting our ability to assess its impact directly in this analysis. These findings suggest that in order to reduce the incidence of missed diagnosis, a thorough examination of the proximal colorectal region is vital, especially the hepatic flexure of the colon and the cecum, which are difficult to operate.

This study has several limitations. It should be noted that our definition of missed CRC may include interval cancers, which could lead to an overestimation of the true miss rate, as lesion-level progression data are not available in the PLCO dataset. The lack of specific lesion location information possibly influenced the estimation of the missed diagnosis rate. Moreover, the PLCO database does not include data on bowel preparation quality, which is a known factor influencing the detection of adenomas and colorectal cancer. This absence may limit the accuracy of estimating missed diagnosis rates. In addition, we defined patients with adenoma or advanced adenoma on baseline or follow-up colonoscopy that progressed to CRC within 3 years and patients with hyperplastic polyps or benign polyps on baseline or follow-up colonoscopy that progressed to CRC within 7.5 years as missed diagnosis cases. However, whether the time standard we set is accurate remains to be determined.

Recent advances in colorectal cancer (CRC) screening technologies, such as multi-target stool DNA testing and blood-based liquid biopsy, have demonstrated encouraging sensitivity and specificity for detecting early-stage CRC and advanced adenomas [19, 20]. These non-invasive methods are less affected by operator variability and bowel preparation quality, which are limitations of traditional colonoscopy. When used in conjunction with colonoscopy, they may offer a complementary approach to improve overall diagnostic performance. In addition, emerging technologies such as artificial intelligence (AI)-assisted colonoscopy and molecular biomarker profiling hold potential to further reduce the missed diagnosis rate, particularly for lesions located in the proximal colon [21].

In conclusion, our study found that the rate of missed CRC diagnosis by colonoscopy is relatively high in the US population, especially in patients with a history of colon polyps, late clinical staging, and proximal CRC. Thus, a careful and comprehensive colonoscopy in these patients and appropriate screening frequency, especially in patients with a history of colonic polyps, are needed to reduce the rate of missed CRC diagnosis. Recently, the improvement in the screening accuracy of new non-invasive CRC screening technologies, such as fecal DNA and liquid biopsy, has overcome the disadvantages of traditional colonoscopy, which include susceptibility to the influence of intestinal preparation and operator’s technical level [22]. Nevertheless, further improvement in the accuracy of detection techniques and an effective combination of new detection techniques and traditional colorectal endoscopy are the new challenges for early CRC screening.

## Data Availability

Data were obtained from the National Cancer Institute’s prostate, lung, colorectal, and ovarian (PLCO) cancer screening trial.
